# In Silico Evaluation of the Effectivity of Approved Protease Inhibitors against the Main Protease of the Novel SARS-CoV-2 Virus

**DOI:** 10.3390/molecules25112529

**Published:** 2020-05-29

**Authors:** Phaedra Eleftheriou, Dionysia Amanatidou, Anthi Petrou, Athina Geronikaki

**Affiliations:** 1 Department of Biomedical Sciences, School of Health, International Hellenic University, 57400 Thessaloniki, Greece; dionusiaam@gmail.com; 2 Department of Pharmaceutical Chemistry, School of Pharmacy, Aristotle University of Thessaloniki, 54124 Thessaloniki, Greece; anthi.petrou.thessaloniki1@gmail.com

**Keywords:** coronavirus, SARS-CoV-2, protease inhibitors, HCV protease inhibitors, DPP-4 inhibitors, a-thrombin inhibitors, docking

## Abstract

The coronavirus disease, COVID-19, caused by the novel coronavirus SARS-CoV-2, which first emerged in Wuhan, China and was made known to the World in December 2019 turned into a pandemic causing more than 126,124 deaths worldwide up to April 16th, 2020. It has 79.5% sequence identity with SARS-CoV-1 and the same strategy for host cell invasion through the ACE-2 surface protein. Since the development of novel drugs is a long-lasting process, researchers look for effective substances among drugs already approved or developed for other purposes. The 3D structure of the SARS-CoV-2 main protease was compared with the 3D structures of seven proteases, which are drug targets, and docking analysis to the SARS-CoV-2 protease structure of thirty four approved and on-trial protease inhibitors was performed. Increased 3D structural similarity between the SARS-CoV-2 main protease, the HCV protease and α-thrombin was found. According to docking analysis the most promising results were found for HCV protease, DPP-4, α-thrombin and coagulation Factor Xa known inhibitors, with several of them exhibiting estimated free binding energy lower than −8.00 kcal/mol and better prediction results than reference compounds. Since some of the compounds are well-tolerated drugs, the promising in silico results may warrant further evaluation for viral anticipation. DPP-4 inhibitors with anti-viral action may be more useful for infected patients with diabetes, while anti-coagulant treatment is proposed in severe SARS-CoV-2 induced pneumonia.

## 1. Introduction

The coronavirus SARS-CoV-2, responsible for the pandemic which started in Wuhan, China, during the latter part of 2019, has spread to 213 countries, areas and territories according to the WHO [1,2] by April 16th 2020 and continues to spread, infecting approximately 84,500 and causing the death of approximately 8000 people daily. Although young people usually develop mild symptoms, it can cause severe lower respiratory disease affecting mostly the elderly and individuals with other co-morbidities such as cardiovascular problems, pre-existing respiratory disease, diabetes, hypertension or cancer [1].

SARS-CoV-2 belongs to the βCoV genera of the *Coronavirinae* subfamily of the order of Nidovirals [3]. Compared to SARS and MERS—coronavirus strains that caused the two most recent epidemics, in 2002–2003 and 2012, respectively—it mostly resembles SARS-CoV-1 (2002–03), with 79.5% sequence identity and the same mechanism for host cell entrance, via the angiotensin-converting enzyme-2 (ACE-2) surface protein of the cell [4].

Due to the urgent need for effective drugs to attenuate the disease, and the long-lasting process of novel drug development, researchers are focusing on “drug repositioning”. Among the drugs approved or developed for other purposes that have been proposed for the treatment of SARS-CoV-2 and are under clinical trials, most are enzyme inhibitors, targeting the RNA-dependent RNA polymerase (RDRP) or the protease of the virus. Among them, favipinavir, ribavirin, remdesivir and galidesivir act as RDRP inhibitors while lopinavir, ritonavir [5,6] and danoprevir are viral protease inhibitors. In addition, drugs aiming to prevent the entrance and formation of the viral particles have been proposed, such as chloroquine, hydroxychloroquine [7] and APN01 [8].

Lopinavir and ritonavir designed and approved for the treatment of AIDS were the first protease inhibitors used for the treatment of SARS-CoV-2 [5,6]. Both drugs were proposed as promising agents for the treatment of SARS-CoV-1 by an in silico study conducted in 2016 which had evaluated the probable activity of several HIV-1 protease inhibitors against the SARS-CoV-1 protease using docking analysis [6]. The in silico results were promising, although coronavirus proteases are cysteine proteases while the HIV-1 protease belongs to the aspartate protease family [6].

Upon release of the first SARS-CoV-2 main protease structure (Mpro) to the Protein Data Bank, on March 2020, several in silico studies have been conducted to find potent protease inhibitors [9,10,11,12,13,14,15,16,17,18,19]. The in silico-evaluated compounds were mostly approved drugs of all categories [9,10,11,12,13,14,15,16,17,18,19], and natural plant [11,13] as well as marine [16] products. Since docking analysis was performed, in most cases, in randomly selected approved drugs, independently of the rational of their initial design and their initial target, pharmaceutical agents of different drug categories have been proposed. Among the most potent predicted inhibitors were anti-bacterial [10,19], anti-fungal [10], anti-viral [12], anti-tuberculosis [12], anti-nimatoid [17], anti-protozoal [17], anti-helminthic [19], anti-inflammatory [19], anti-tumor [10,12], vasodilator [10] vasoprotective [12], anti-convulsant [12], bronchodilator [12], anti-psychotic [10] agents and vitamins [12]. Among the anti-viral agents are ribavirin, a nucleoside analogue used for the treatment of Lassa fever virus, influenza A and B and HCV [12]; telbivudine, a nucleoside analogue used for the treatment of HAV [12]; the HIV-1 integrase inhibitors raltegravir and dolutegravir [13] and the HIV-1 protease inhibitors, darunavir and saquinavir [14]. According to a more recent in silico research, published on April 15th, the HCV protease inhibitors, simeprevir and faldaprevir as well as the HIV-1 protease inhibitors indinavir and tipranavir along with saquinavir were predicted to act as better Mpro inhibitors compared to the lopinavir and ritonavir used in clinical practice [15]. Danoprevir was the first anti-HCV protease inhibitor which was approved for clinical trials (clinical trial: NCT04291729).

Proteases are central enzymes in the biology of humans and viruses, and several of them have become drug targets for anti-viral therapy as well as for the treatment of various diseases. There is a number of approved drugs that act as protease inhibitors [20]. Among the main proteolytic enzymes are the viral proteases of HIV-1, with more than fifteen approved or on-trial drugs, and of HCV, with more than ten drugs. In addition, a number of drugs against human proteases have been developed. Among them are renin inhibitors, with seven compounds and ACE inhibitors, with more than twenty compounds with applications in the treatment of hypertension; thrombin as well as coagulation Factor Xa inhibitors used as anti-thrombotic agents. DPP-4 inhibitors, gliptins, are used for the treatment of type II diabetes, with seventeen drugs of this type mentioned in the WHO drugs stem book [21].

The above proteases belong to different classes according to the catalytic amino acid. While the SARS-CoV-2 main protease is a cysteine protease [20,22], the HIV-1 enzyme is an aspartate protease [20,23]. The human drug target renin also belongs to this family. On the other hand, the HCV protease [24] as well as the human protease DPP-4 [25], the coagulation Factor Xa [26] and thrombin [27] are serine proteases, while the angiotensin-converting enzyme (ACE) belongs to the metalloproteinase family [20,28].

As far as cleavage recognition motifs are concerned, the SARS-CoV-2 main protease cleaves the viral polyprotein at no less than 11 sites, recognizing the sequence Leu-Gln ↓ Ser/Ala/Gly [22]. The HIV-1 protease recognizes nine cleavage sites in its polyprotein substrate, with sequence differences that keep the feature of cleavage, most commonly, between Phe and Pro. Alternatively, it cleaves between amino acids with hydrophobic or aromatic side chains (Tyr/Met/Leu/Phe ↓ Pro/Phe/Tyr/Ala/Leu/Met) [23]. The HCV protease cleaves its polyprotein substrate at four sites, recognizing a Glu/Asp-Glu/Asp-XXX-Cys ↓ Ser general motif [24]. The human organism protease DPP-4 preferentially cleaves at X ↓ Pro or X ↓ Ala sites [25], whereas thrombin preferentially cleaves at the Gly/Ala/Val/Ile/Pro-Arg ↓ Ser/Ala/Gly/Thr/(notE/D)-X(notE/D)-Arg/(notE) motif [27]. The coagulation Factor Xa cleaves after Arg, recognizing a motif Val/Pro/Ile-Gln/Asp/Glu-Phe/Gly-Arg ↓ Ser-Leu [26]. Renin recognizes the sequence Ile-His-Pro-Phe-His-Leu↓ [29], while angiotensin-converting enzyme (ACE) recognizes the angiotensin I sequence Asp-Arg-Val-Tyr-Ile-His-Pro-Phe ↓ His-Leu [28].

Similarities between proteases at the catalytic amino acids of the active site and in the nature of amino acids of the substrate may be an indication for efficient inhibition of the proteases by the same inhibitors. However, the nature of surrounding amino acids of the active site and the 3D structure of the active center are also of great importance.

In the present study, the 3D structure of the SARS-CoV-2 main protease was compared with the 3D structures of proteases which are drug targets with approved inhibitors. In addition, a number of approved inhibitors of the above proteases were evaluated by docking analysis for their ability to inhibit the SARS-CoV-2 main protease, interacting with the active site of the enzyme.

## 2. Results and Discussion

### 2.1. The 3D Structure Protein Alignment

Since the 3D structure of the active site of the enzyme is crucial for catalytic activity, we proceeded to a comparison of the SARS-CoV-2 main protease, Mpro, with the HIV-1 protease, the HCV protease (NS3 protein) and the human proteases DPP-4, thrombin, Factor Xa, renin and ACE, which constitute known drug targets with approved inhibitors.

**Figure 1:**
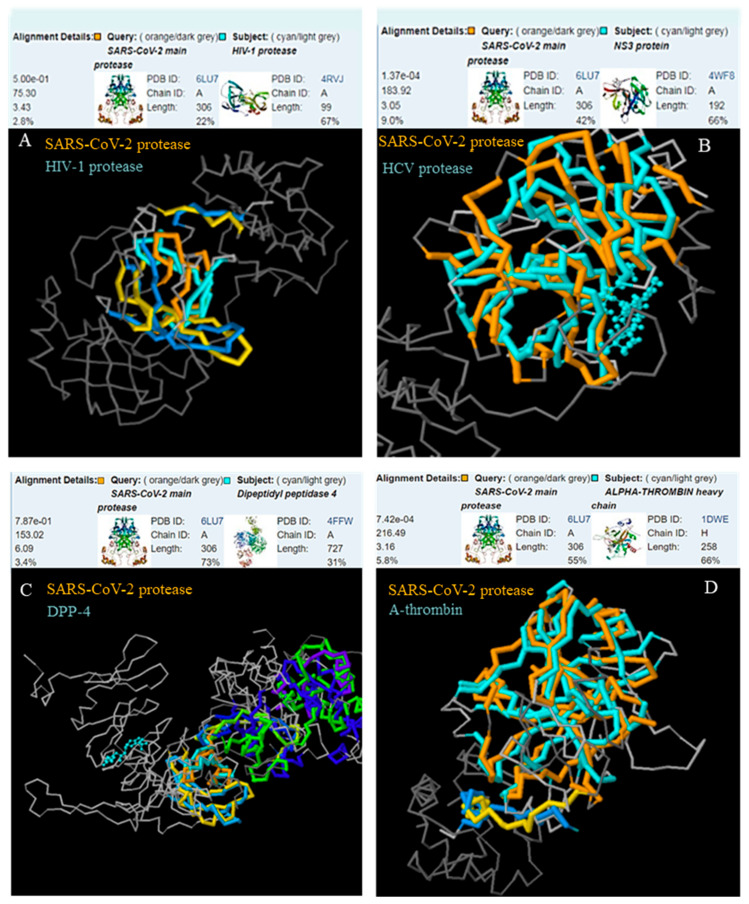
The 3D structural alignment between the SARS-CoV-2 main protease and the HIV-1 protease (**A**), the HCV protease (**B**), DPP-4 (**C**) and α-thrombin (**D**).

**Figure 2:**
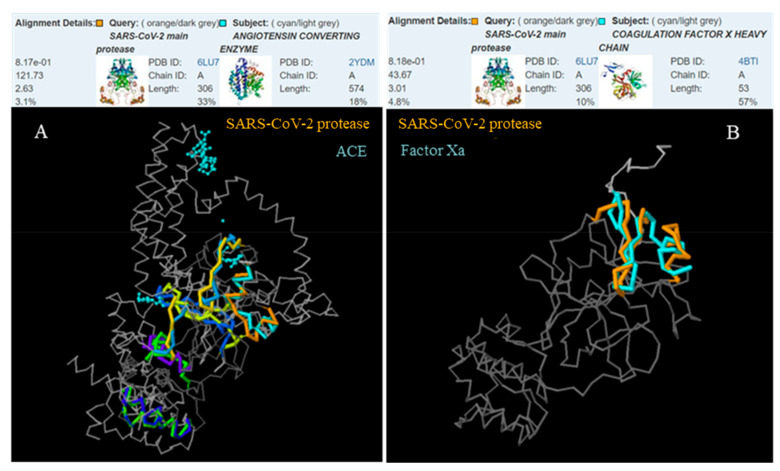
The 3D structural alignment between the SARS-CoV-2 main protease and the angiotensin-converting enzyme (**A**), and the coagulation Factor Xa heavy chain (**B**).

Significant 3D similarity was found between the SARS-CoV-2 and the HCV protease (*p* = 1.34 × 10^−4^) as well as between the SARS-CoV-2 protease and α-thrombin (*p* = 7.42 × 10^−4^). Interestingly, the protein parts with increased structural alignment included the catalytic area of the enzyme. The lowest similarity was observed in the case of angiotensin-converting enzyme and coagulation Factor Xa (Figure 1 and Figure 2).

The structural similarity between the SARS-CoV-2 protease and some of the selected proteases, in combination with the existence of the same amino acids at certain positions of the substrate cleavage site, such as Ser at the P1’ position of the recognition sequence of the HCV protease and thrombin are promising features in the effort to identify effective SARS-CoV-2 protease inhibitors among the approved drugs of the selected proteases.

To investigate this possibility further, we proceeded to docking analysis of randomly selected approved or on-trial inhibitors of the selected proteases (Table 1). An exception from the random selection was made for the HIV-1 inhibitors where lopinavir and ritonavir were selected because they have already been proposed for the treatment of SARS-CoV-2. The rest of the drugs that were selected are the anti-HCV drugs telaprevir and boceprevir, the DPP-4 inhibitor sitagliptin, the renin inhibitor alisliren, the ACE inhibitor captopril, the thrombin direct inhibitors argatroban and dabigatran and the coagulation Factor Xa inhibitor rivaroxaban.

### 2.2. Docking Analysis

Docking analysis was performed using the SARS-CoV-2 structure 6LU7. The results were promising for most of the compounds, with the exception of the ACE and renin inhibitors, captopril and aliskiren, for which the calculated free binding energy was −5.17 and −4.66 kcal mol^−1^, respectively (Table 2). According to our previous experience and to the literature, a free binding energy higher than −5.5 kcal mol^−1^ is an indication that the compound is inactive [32,33].

The best results were obtained for the HCV protease inhibitors telaprevir and boceprevir, with free binding energies of −10.05 and −9.16 kcal mol^−1^, respectively, followed by the thrombin inhibitor argatropan and the DPP-4 inhibitor, sitagliptin, with free binding energies of −9.03 and −8.80 kcal mol^−1^, respectively. The HIV-1 protease inhibitors ritonavir and lopinavir showed a little lower predicted activity, with free binding energies of −8.96 and −8.65 kcal mol^−1^, respectively. Docking analysis to the whole enzyme indicated that the enzyme active site is the second preferred binding site of lopinavir, ritonavir and boceprevir. According to this observation, the expected inhibitory action of these compounds is to be lower than that predicted based on their binding energy to the active site [32]. Rivaroxamban and dabigatran are also expected to be active, with calculated free binding energies of −7.97 and −6.57 kcal mol^−1^, respectively.

Since the evaluation of the randomly selected HCV protease, DPP-4, α-thrombin and coagulation Factor Xa inhibitors showed promising results, we proceeded to the evaluation of a more extended sample of approved drugs of these categories, including a number of drugs currently in phase III clinical trial (Table 3, Table 4). For better estimation of the probable inhibitory effect of the selected compounds, a second protein structure of the SARS-CoV-2 protease was also used (PDB ID: 6M2N). The whole enzyme, including both protease subunits, was selected for the process. For comparison reasons, the estimated binding energy of the compounds to their initial targets were also calculated. For the same reasons, for some of the most promising SARS-CoV-2 candidate inhibitors, the estimated binding energy to the HIV-1 4RVJ protease structure was calculated.

Docking analysis of both SARS-CoV-2 protease structures gave similar results. Interestingly, 20 out of the 24 compounds which were selected for evaluation exhibited lower estimated binding energy than the initial inhibitor of the 6M2N structure (−7.45 kcal mol^−1^) [34] and of the N3 inhibitor (−7.41 kcal mol^−1^) [35]. Docking of the compounds to the target proteases for which they were designed showed a generally higher binding probability (lower estimated binding energy) for the initial targets compared to the SARS-CoV-2 protease but the most active compounds showed comparable results for the two targets. For example, faldaprevir exhibited an estimated binding energy of −11.27 kcal mol^−1^ for the SARS-CoV-2 protease and −11.15 kcal mol^−1^ for the HCV protease, while teneligliptin showed −9.87 kcal mol^−1^ for the new protease and −9.16 kcal mol^−1^ for its initial target.

According to the estimated free binding energies, all evaluated drugs could act as SARS-CoV-2 protease inhibitors. Among the HCV protease inhibitors, Faldaprevir exhibited the lowest free binding energy.

Teneligliptin and gemigliptin are the most promising drugs among the DPP-4 inhibitors. In the group of anti-coagulants, inogartan and argatroban of the first randomly selected compounds were the most promising among a-thrombin inhibitors and edoxaban among Factor Xa inhibitors. Docking analysis of the whole enzyme indicated that the enzyme active site is the first preferred binding site for all the HCV protease and DPP-4 inhibitors presented in Table 3 but the third preferred site for inogatran and the second for edoxaban. Consequently, the expected inhibitory action of inogatran and edoxaban are to be lower than that predicted based on their relative binding energy to the active site. In conclusion, faldaprevir, teneligliptin and gemigliptin are the compounds expected to exhibit the highest activity against the SARS-CoV-2 main protease.

The SARS-CoV-2 protease preferentially cleaves its substrate after Gln which follows Leu and before a Ser or Ala or Gly amino acid (Leu-Gln ↓ Ser/Ala/Gly). The Gln amino acid, at the P1 position of the substrate, is an amino acid with a relatively long side chain leading to a polar amide group with hydrogen donor/acceptor possibility. As shown by studies of complexes of the Mpro enzyme with substrate analogues [34], Gln is placed in vicinity to Cys145, at the subsite S1 of the active center, surrounded by the side chains of the amino acids Asn142, Glu166, His163, and His172 and the main chains of Phe140 and Leu141. The P2 amino acid, Leu, has a relatively long, hydrophobic but not bulky or aromatic side chain. The side chain of Leu is inserted at the S2 subsite, which consists of the side chains of the amino acids His41, Met49, Tyr54 and Met165 placed in vicinity to Asp187. The P4 amino acid of the peptide interacts with the amino acids Met165, Leu167, Phe185, and Gln192 and the main chain of Gln189.

In the case of the substrate analogue N3, in addition to polar or hydrophobic interactions, a hydrogen bond (Hb) is formed between the His163 side chain and O of the lactam ring which serves as a mimetic of the Gln amino acid of the natural substrate [34]. A second Hb is formed between the H of the closest to the lactam ring NH group of the backbone of the ligand and the CO of the main chain of His164 of the enzyme, while the NH group of the backbone of Leu amino acid of the ligand interacts with the CO of the backbone of Gln189 of the enzyme. The lactam ring as a mimetic of the Gln amino acid of the substrate is also present at the structure of the a-ketomide inhibitors designed by Zang et al. [22]. In this case, the H of the NH group of the lactam ring forms two hydrogen bonds with the CO of the main chain of Phe140 and the carboxylate of Glu166, while the CO oxygen of the lactam moiety forms a hydrogen bond with the imidazole of His163. In the most active compound of this series, the P2 Leu of the natural substrate has been replaced by a cyclopropane, while the pyridon ring at the P3 position participates in complex stabilization through hydrogen bonds with the O of the side chain of Gln189 and the NH of the main chain of Glu166.

The two above substrates constitute peptidomimetics with peptide bonds between amino acid mimetic moieties. In both cases, the catalytic amino acid Cys145 forms a covalent bond with the ligand. Most of the approved drugs proposed as SARS-CoV-2 protease inhibitors according to in silico studies are not peptidomimetics. According to docking analysis, various groups may be placed at the S1 subsite such as the 7-methoxy-8-methyl-quinoline moiety of simeprevir [15] or the oxadiazole group of raltegravir, which adopts a curved form within the binding pocket [13]. Interaction with Thr24, Thr25 and Ser46 also seems to play an important role in complex stabilization of many compounds including raltegravir and ribavirin [12].

Most inhibitors used in this study do not contain the classic peptide bonds but share with peptide substrates the characteristic of many polar, Hd/a groups.

**Figure 3:**
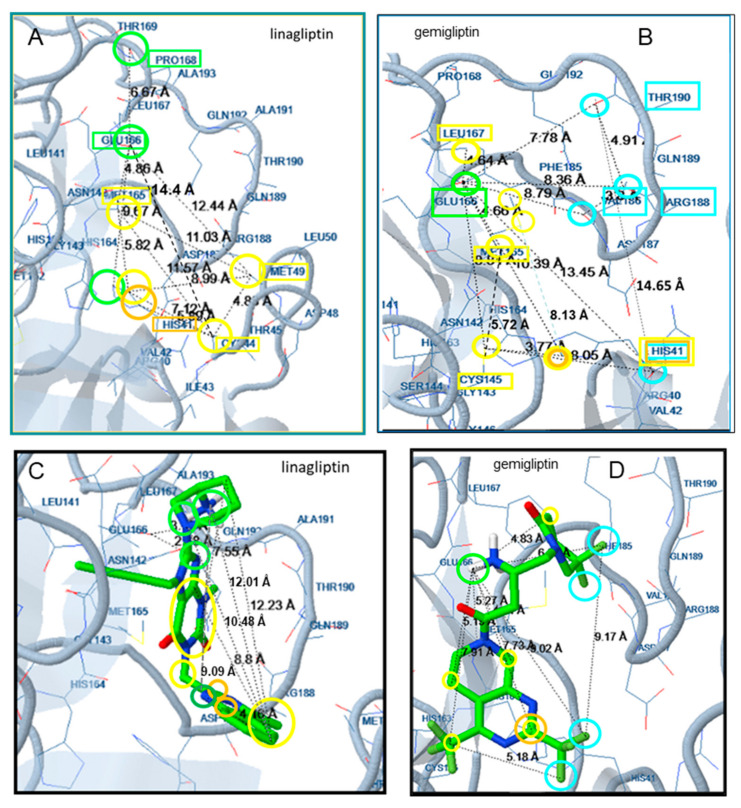
Docking of linagliptin (**A**,**C**) and gemigliptin (**B**,**D**) at the SARS-CoV-2 structure 6M2N. Distances between the interacting amino acids are shown in (**A**,**B**). Distances within the molecule of the inhibitors are shown in (**C**,**D**). Colors indicate participation in hydrophobic interactions (yellow), pi interactions (dark yellow), hydrogen bonds (light green, polar interactions (dark green), and halogen bonds (blue).

In gemigliptin, the tetrahydropyridine ring and the 2-amino-4-oxobutyl bridge are placed at the S1 subsite between Glu166 and Cys145. A hydrogen bond between the CO group of the backbone of Glu166 and the NH_2_ group of the 2-amino-4-oxobutyl bridge contributes to complex stabilization, while Cys145 participates in hydrophobic interactions with carbons of the bicyclic moiety (Figure 3). The trifluoromethyl pyrimidine moiety interacts with His41 of the S2 subsite which stabilizes the complex by halogen bond formation with the F atoms and pi interactions with the pyrimidine ring. Halogen bonds are also formed between the F atoms of the fluoropiperidinone ring and amino acids Val186, Arg188 and Thr190.

In linagliptin, the N of the piperidine ring participates in hydrogen bond interactions with the O of the CO group of the main chain of Glu166 of the S1 subsite, while the NH_2_ substituent of the ring forms Hb with Pro168. The His41 of the S2 subsite participates in complex stabilization by polar interaction with the N atom of the methylquinazoline and by hydrophobic and pi interactions with the carbons of this moiety, while Met49 also forms hydrophobic interactions. Additional stabilization is achieved by the interaction of Met165 with the dihydropurine moiety (Figure 3).

**Figure 4:**
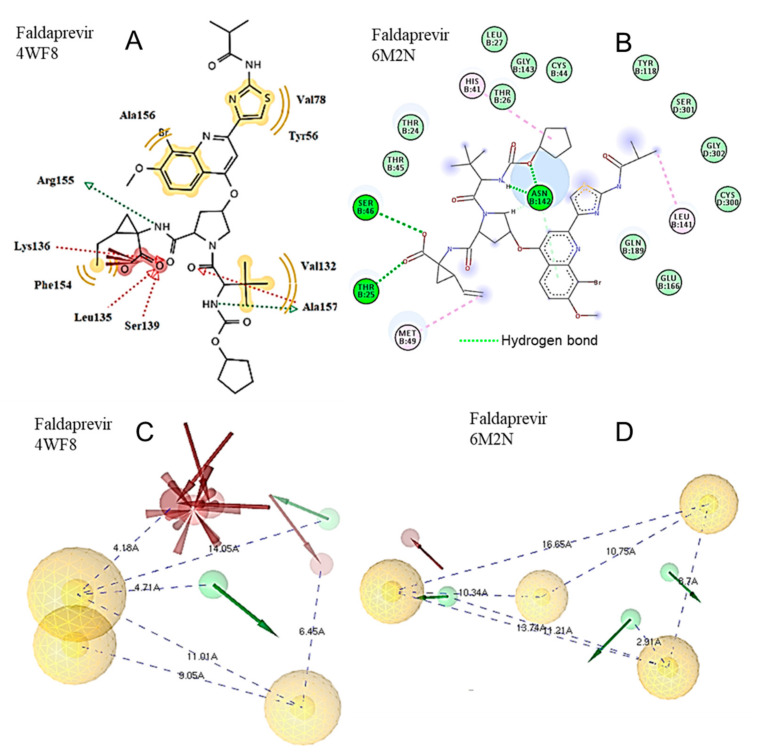
Docking of faldaprevir to the HCV protease structure 4WF8 (**A**) and to the SARS-CoV-2 protease structure 6M2N (**B**). The main hydrophobic (yellow balls) and hydrogen donor (green balls with arrows) and acceptor (red balls with arrows) moieties of the molecule participating in complex stabilization with the HCV protease structure 4WF8 and the SARS-CoV-2 protease structure 6M2N are shown in (**C**,**D**), respectively.

In faldaprevir, the cyclopentyl carbamate moiety participates in hydrogen bond formation with the Asn142 of the S1 subsite, while hydrogen bonds are also formed between the 2-ethenylcyclopropane-1-carboxylic acid group and the amino acids Thr25 and Ser46 (Figure 4).

**Figure 5:**
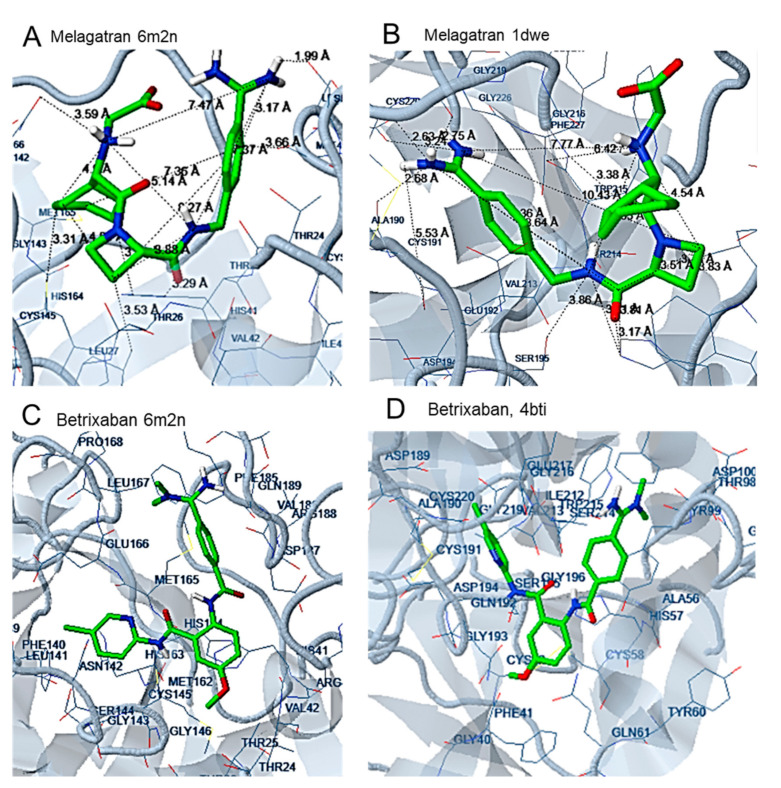
Docking of melagatran to the Mpro structure 6M2N (**A**) and to the a-thrombin structure 1DWE (**B**). Docking of betrixaban to the Mpro structure 6M2N (**C**) and to the Factor Xa structure 4BTI (**D**).

In the case of melagatran, a curved conformation is adopted. The catalytic amino acid, Cys145, forms hydrophobic interactions with cyclohexan, while Asn144 of the S1 subsite strongly contributes to complex stabilization via polar interaction with the *N* atom of the aminoacetyl acid group. At the S2 subsite, the NH group of the side chain of His41 participates in hydrogen interaction with the O of the methyl carbamoyl bridge, while Met49 is involved in hydrophobic interactions with the phenyl ring of the molecule. In addition, the OH group of the nearby Ser46 participates in hydrogen bond formation with the NH_2_ of the benzimidamide group (Figure 5).

In betrixaban, the chloropyridin moiety is placed at the S1 subsite, interacting via halogen bonds with the amino acids Glu166, Phe140 and Leu141 and via hydrophobic interactions with Cys145. In addition, a hydrogen bond is formed between Cys145 and the *N* of the amide bridge connecting the chloropyridin-2yl and the methoxybenzyl rings of the molecule. His41 of the S2 subsite forms pi interactions with the carbons of the methoxyphenyl ring. The *N*,*N*-dimethylbenzimidamide moiety, placed in the area of the S4 subsite, strongly contributes to complex stabilization via the hydrophobic interaction between Met165 and the phenyl ring as well as by hydrogen bond formation between the amino acids Arg188, Thr190 and Gln192 and the *N* of the *N*,*N*-dimethylbenzimidamide moiety (Figure 5).

In general, there is a great variety between the groups placed at the S1 or S2 subsites of the enzyme, as concluded by our results and the literature [12,13,15]. Most of the amino acids involved in complex stabilization of the natural substrate and the peptide analogues [22,34] participate in interactions with the studied compounds as well. However, the substantial interaction with His163 in peptidomimetic complexes is not observed in the interaction pattern of the most potent compounds. Different structures may offer the possibility of interaction with the crucial amino acids of the SARS-CoV-2 active site contributing to complex stability. However, the right distances between polar, Hd/a, hydrophobic/aromatic moieties constitute important features of each structure.

**Figure 6:**
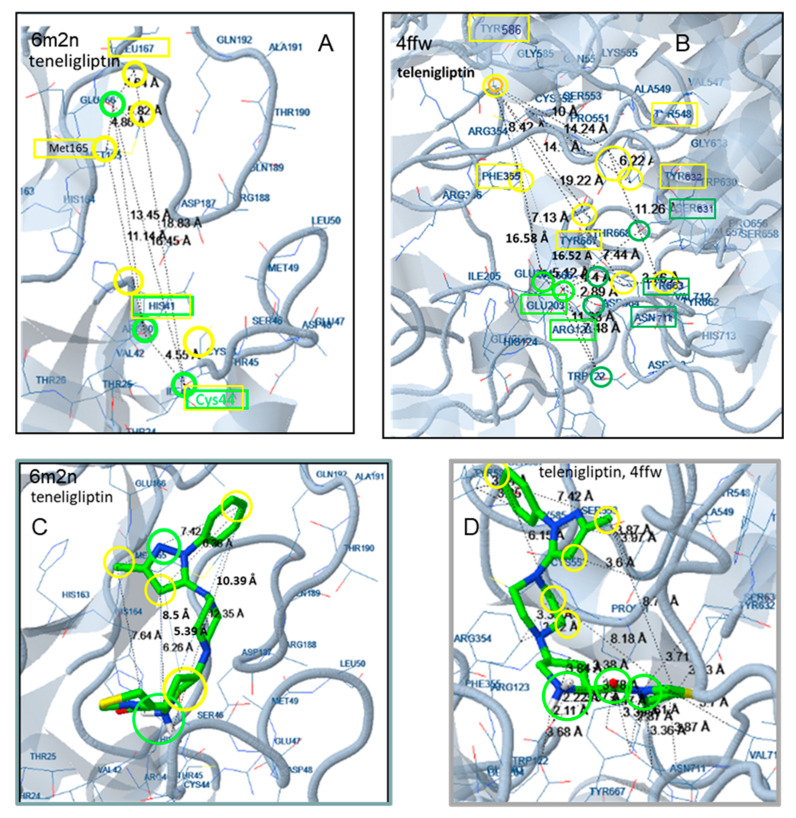
Docking of teneligliptin to the Mpro structure 6M2N (**A**,**C**) and to the DPP-4 structure 4FFW (**B**,**D**). Distances between the interacting amino acids are shown in (**A**,**B**). Distances within the molecule of the inhibitor are shown in (**C**,**D**). Colors indicate participation in hydrophobic interactions (yellow), pi interactions (dark yellow), hydrogen bonds (light green, polar interactions (dark green), and halogen bonds (blue).

**Figure 7:**
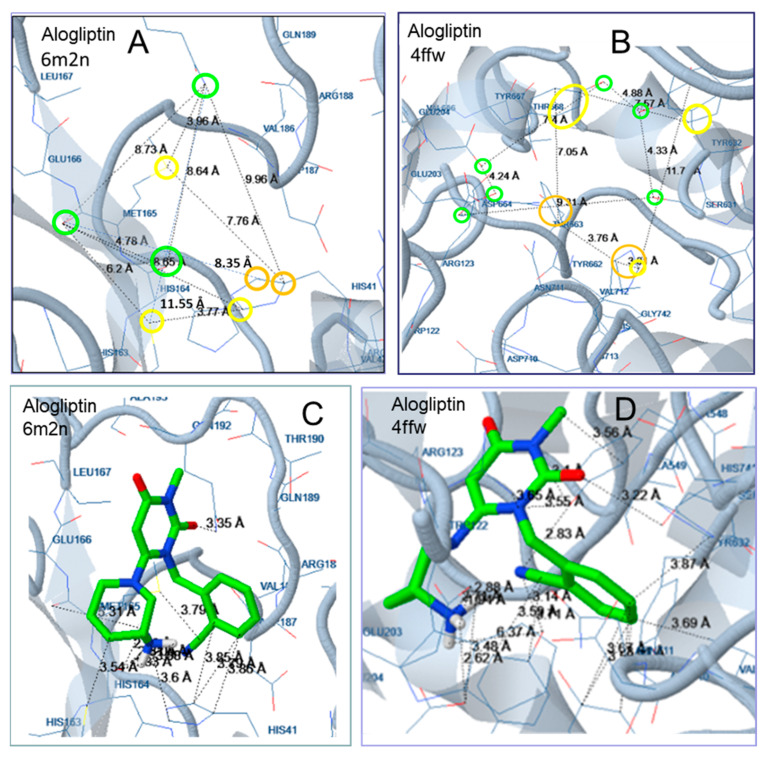
Docking of alogliptin to the Mpro structure 6M2N (**A**,**C**) and to the DPP-4 structure 4FFW (**B**,**D**). Distances between the interacting amino acids are shown in (**A**,**B**). Distances within the molecule of the inhibitor are shown in (**C**,**D**). Colors indicate participation in hydrophobic interactions (yellow), pi interactions (dark yellow), hydrogen bonds (light green), polar interactions (dark green), and halogen bonds (blue).

According to docking analysis, all active molecules are long enough to interact simultaneously with amino acids surrounding Glu166, Cys145 and amino acids in the vicinity of Thr25 and His41 and contain several hydrophobic and aromatic moieties as well as hydrogen bond donor/acceptor groups capable of hydrogen bond formation and polar interactions. The enzyme amino acids involved in hydrogen bond formation are Glu166, Pro168, Gln189, Arg188, Thr24, Thr25, Thr26, His41, Cys44, Ser46, Asn142, Gly143, Cys145, His164, Thr190, and Glu192 (Figure 3, Figure 4, Figure 5, Figure 6 and Figure 7). Apart from the polar and hydrophobic interactions, at least two hydrogen bonds are formed in the case of the most active compounds, while interactions with Phe140 were also involved in complex stabilization of argatroban.

The most promising compounds of all categories contain multiple hydrophobic moieties as well as polar/hydrogen donor/acceptor moieties. The most active molecules of the categories of gliptines and previrs are relatively long molecules and adopt linear conformations.

Hydrophobic moieties at distances of approximately 9 Å, 11 Å, 14 Å and 16 Å are present in all potent compounds. Met165, His41, Cys44, Cys145, Leu167 of the Mpro active site are mainly involved in hydrophobic and pi interactions, with a maximum distance between them of approximately 18 Å (Figure 3, Figure 4, Figure 5, Figure 6 and Figure 7). Polar or hydrogen donor/acceptor moieties are present at multiple distances, with a distance of 8 Å being common in active compounds. Glu166 and His 41, at a distance of approximately 13 Å, participate in hydrogen bond and polar interactions of the most potent gliptins (Figure 3 and Figure 6), while Ser46, Thr25 and Asn142, at a distance of approximately 12 Å, participate in hydrogen bond formation of faldaprevir. In the SARS-CoV-2 protease, amino acids involved in hydrophobic and polar interactions are distributed in all the active sites, although pi interactions are mostly observed at the region of His41. Distances of approximately 5 Å, 9 Å, 11 Å and 16 Å are observed between amino acids participating in polar and hydrophobic interactions.

Amino acids with hydrophobic or polar/hydrogen donor acceptor properties exist at similar distances at the active sites of the initial target of these compounds, DPP-4 and the HCV protease, respectively.

For example, in the case of the gliptins’ initial target DPP-4, a great number of tyrosine amino acids are present at the active site. As shown in the case of teneligliptin, Figure 6, the amino acids Tyr586, Phe355, Tyr667, Tyr667, Ty548, and Tyr632 are involved in hydrophobic and pi interactions with distances among them varying between 8 Å and 19 Å. The amino acids Arg123, Glu203, Glu204, Ser631, Tyr633 and Asn711 are involved in polar interactions and hydrogen bond formation. All hydrophobic interactions originate from amino acids oriented at the same moiety of the active site, while the amino acids participating in polar interaction are placed in the other moiety. Distances, between 11 and 18 Å are observed between amino acids of the active site participating in polar and hydrophobic interactions. The length of the molecules, the great number of polar and hydrophobic groups, the flexibility of the molecules and the appropriate distances between characteristic groups seem to favor binding of gliptins at the Mpro active site. A higher binding energy was estimated for the smaller molecule alogliptin (Figure 7).

In the case of thrombin inhibitors, a number of hydrogen bonds and hydrophobic interactions mainly stabilize the complex. His41, Ser46 and Met49 participate in hydrogen bond formation between melagatran and Mpro enzyme, while Leu27, His41, Met49 and Cys145 are involved in hydrophobic interactions. Although these interactions lead to a substantially low estimated binding energy of the compound, the significantly higher hydrogen bonds, pi bonds, polar and hydrophobic interactions may be responsible for the much better prediction results for binding to the initial protease target. Ala189, Ala190, Gly219, Cys191, Cys220, Trp215, His 57 and Leu99 seem to be involved in binding to thrombin.

The evaluated compounds were approved drugs, currently on the market with the exception of four compounds which were under phase III trial (Table 4). There are several compounds among the most promising ones, which are characterized as well-tolerated or having no side effects, such as danoprevir and sovaprevir of the HCV protease inhibitors, teneligliptin and gemigliptin but also trelagliptin, evogliptin and gosogliptin among the DPP-4 inhibitors, inogatran and melagatran of α-thrombin inhibitors.

The DPP-4 inhibitors developed for the treatment of diabetes type II as well as the α-thrombin and Factor Xa inhibitors, used as anti-coagulants, may not be appropriate for patients who do not suffer such problems. However, they may be worth consideration for patients already being treated for type II diabetes or with anti-coagulants. Moreover, higher platelet count and increased coagulation features are observed in COVID-19 patients with severe pneumonia compared to patients with non SARS-CoV-2-induced pneumonia. Furthermore, according to recent results, anti-coagulant treatment by heparin administration reduced mortality of COVID-19 patients with severe pneumonia and markedly elevated D-dimers [35].

In general, the results can be considered as promising for some of the evaluated drugs and could be useful for scientists working in the field. In vitro evaluation is also within our future targets if the recombinant SARS-CoV-2 protease becomes available in the market.

## 3. Experimental Part

### 3.1. The 3D Structure Protein Alignment

The 3D alignment was performed between the 3D structure of the SARS-CoV-2 main protease (PDB ID:6LU7) and (a) the HIV-1 protease (Protein Data Bank ID: 4RVJ), (b) the HCV protease, NS3protein (PDB ID:4WF8), (c) dipeptidil transpeptidase-4, DPP-4 (PDB ID:4FFW), (d) α-thrombin (PDB ID:1DWE), (e) coagulation Factor Xa (PDB ID: 4BTI) and (f) ACE-2 (PDB ID:2YDM) using the RCSB Protein Data Bank 3D structure protein comparison tool (jFATCAT flexible method) [36,37].

### 3.2. Docking Analysis

Docking analysis was carried out on a molecular docking server using Autodock [38,39], as previously described [32]. The Lamarckian genetic algorithm (LGA) and the Solis and Wets local search method [40] were used for the docking simulation. During docking, all rotatable torsions were released. AutoDock parameter set- and distance-dependent dielectric functions were used in the calculation of the van der Waals and the electrostatic terms, respectively—100 different runs that were set to terminate after a maximum of 2,500,000 energy evaluations were performed in each docking experiment. The population size was set to 150. A translational step of 0.2 Å, and quaternion and torsion steps of 5 were applied during the search. Docking analysis of the HCV protease and the HIV-1 protease presented in Table 3 was performed using Autodock4.2. via Ligandscout. Among the values calculated by the program is the estimated free binding energy, which is an indicator of the probability of the compounds to form a stable complex with the selected enzyme target and consequently the probability to be effective enzyme inhibitors.

The protein structures chosen from the Protein Data Bank and the docking center and docking box chosen for each docking analysis were as follows: for docking analysis of the SARS-CoV-2 main protease enzyme, the structure 6LU7 of the enzyme in a complex with the competitive inhibitor N3 (xavlxx: *N*-[(5-methylisoxazol-3-yl)carbonyl]alanyl-L-valyl-L-*N*~1~-((1R,2Z)-4-(benzyloxy)-4-oxo-1 -{[(3R)-2-oxopyrrolidin-3-yl]methyl}but-2-enyl)-L-leucinamide) [34,35] was used. This was the first enzyme structure in complex with an inhibitor that was released in the Protein Data Bank on March 18th, 2020 and was the only one available when the preliminary docking studies of this research were performed. Although the enzyme is in the form of a dimer of identical subunits, only one subunit was available in the form of a PDB file at the PubMed structure. The docking center was kept at x = −10.46, y = 4.1037, and z = 73.009 and the docking box was set at x = 30, y = 40, and z = 25 to surround the binding site of the inhibitor (active site of the enzyme).

For active compounds, the docking was repeated, extending the docking box to cover the whole enzyme, to ensure that the compound does not preferentially bind to other sites of the enzyme instead of the active site [32].

For better estimation of the probable inhibitory effect of the selected compounds, a second protein structure of the SARS-CoV-2 protease complexed with the inhibitor 5,6,7-trihydroxy-2-phenyl-4H-chromen-4-one was also used (PDB ID: 6M2N). In this structure, both subunits were included in the PDB file and the whole enzyme constructed by the two identical subunits was used. The use of the two subunit form was preferred as it could enable the evaluation of a probable interference of the second subunit in binding of the inhibitors, since Glu166 which commonly participates in complex stabilization interacts with Ser1 of the other protein chain. The docking center was set at x = −61.73, y = −35.18, z = 23.26 and the docking box was set at x = 20, y = 20, z = 20.

For comparison reasons, the estimated binding energy of the compounds to their initial targets was also calculated. The structures chosen were the HCV structure 2WF8 in complex with asunaprevir (center: x = 38.317, y = 14.227, z = 18.86, box x = 35, y = 40, z = 30), the DPP-4 structure 4FFW in complex with sitagliptin(center: x = 20.71, y = −9.22, z = 53.53, box x = 25, y = 25, z = 25), the a-thrombin structure 1DWE in complex with the inhibitor D-phenylalanyl-*N*-[(2s,3s)-6-{[amino(Iminio)methyl]amino}-1-chloro-2-hydroxyhexan-3-Yl]-L-prolinamide (center: x = 32.4, y = 14.07, z = 24.65, box x = 20, y = 20, z = 20) and the coagulation Factor Xa structure 4BTI in complex with the inhibitor 5-chloro-thiophene-2-carboxylic acid [(S)-2-[2-difluoromethoxy-3-(2-oxo-piperidin-1-yl)-benzenesulfonylamino]-3-((S)-3-dimethylamino-pyrrolidin-1-yl)-3-oxo-propyl]-amide (center: x = −11.42, y = −7.062, z = −21.28, box x = 20, y = 20, z = 20).

For some of the most promising SARS-CoV-2 candidate inhibitors, the estimated binding energy to the HIV-1 protease 4RVJ structure (crystallized in complex with amprenavir) was evaluated (center: x = −1.935, y = 4.006, z = 48.913, box x = 40, y = 40, z = 30).

**Figure 8:**
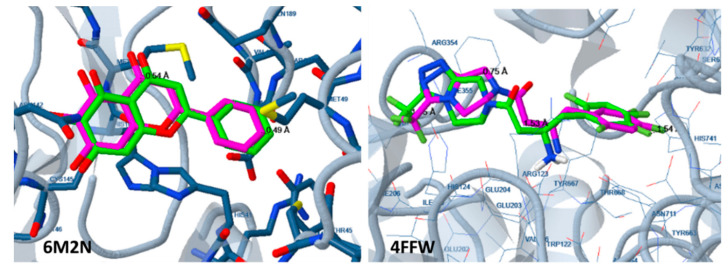
Docking of the initial ligand (5,6,7-trihydroxy-2-phenyl-4H-chromen-4-one) of the 6M2N complex to the SARS-CoV-2 protease structure 6M2N and of the initial ligand (sitagliptin) to the 4FFW structure of DPP-4. The position of the docked ligand (green) is very close to that of the initial ligand (magenta).

In all cases, during enzyme preparation, the inhibitor of the initial enzyme complex was abstracted, and the pH was set at 7.0 for ligand preparation. For the evaluation of the docking method, the initial ligand was docked to the enzyme and the position was compared with its initial position at the complex. Examples of the similarity of the two positions are shown at Figure 8.

## 4. Conclusions

The 3D structure alignment of the SARS-CoV-2 main protease with the HIV-1 and HCV viral proteases as well as with the human proteases DPP-4, thrombin, coagulation Factor Xa, renin and ACE showed greater similarity with the HCV protease and with thrombin. This finding, in combination with the fact that these two proteases preferentially cleave before Ser like SARS-CoV-2 protease, enhances the possibility of finding effective SARS-CoV-2 inhibitors among the approved anti-HCV protease and anti-thrombin inhibitors. Docking analysis of 34 approved or phase III trial protease inhibitors to the SARS-CoV-2 protease revealed several drugs that may act as SARS-CoV-2 protease inhibitors, within the classes of the HCV protease, DPP-4, α-thrombin and coagulation Factor Xa inhibitors. Twenty five of them exhibited estimated binding energy lower than the reference inhibitor N3, and twenty one lower than −8.00 kcal mol^−1^. Several of the most promising compounds are approved drugs characterized as well tolerated in subjects. Compared to lopinavir and ritonavir, most of the evaluated compounds showed better results, as characterized by lower predicted free binding energy and high preference for binding to the active site compared to other sites of the enzyme. These promising in silico results encourage further evaluation for in vitro and in vivo anti-viral activity. The DPP-4 inhibitors developed for the treatment of type II diabetes, as well as α-thrombin and Factor Xa inhibitors used as anti-coagulants, may not be appropriate for patients who do not suffer such problems but may be useful for patients who need such treatment. It is worth mentioning that diabetic patients belong to the high-risk group for this virus, while anti-coagulant therapy has been proposed for COVID-19 patients with severe pneumonia.

## Figures and Tables

**Table 1:** Approved and under-trial protease inhibitors.

Protease	Inhibitors
**HIV-1 Protease**	amprenavir, atazanavir, brecanavir, darunavir, droxinavir, fosamprenavir, indinavir, lasinavir, lopinavir, mozenavir, nelfinavir, palinavir, ritonavir, saquinavir, telinavir, and tipranavir
**HCV Protease**	asunaprevir, boceprevir, ciluprevir, danoprevir, faldaprevir, narlaprevir, neceprevir, simeprevir, sovaprevir, telaprevir, vaniprevir, and vedroprevir
**Dipeptidyl Peptidase-4 (DPP-4)**	alogliptin, anagliptin, bisegliptin, carmegliptin, denagliptin, dutogliptin, evogliptin, gemigliptin, gosogliptin, linagliptin, melogliptin, omarigliptin, saxagliptin, sitagliptin, teneligliptin, trelagliptin, and vildagliptin
**Renin**	aliskiren, ciprokiren, ditekiren, enalkiren, remikiren, terlakiren, and zankiren
**ACE**	alacepril, benazepril, captopril, ceronapril, cilazapril, delapril, enalapril, fosinopril, idrapril, imidapril, indolapril, libenzapril, lisinopril, moexipril, moveltipril, orbutopril, pentopril, perindopril, pivopril, quinapril, ramipril, rentiapril, spirapril, temocapril, trandolapril, utibapril, zabicipril, and zofenopril
**Thrombin**	direct inhibitors: argatroban, inogatran, melagatran and its pro-drug ximelagatran, and dabigatran [30,31]
**Coagulation Factor Xa**	rivaroxaban, apixaban, betrixaban, darexaban, edoxaban, otamixaban, and letaxaban

**Table 2:** Free binding energies of the drugs to the SARS-CoV-2 main protease structure 6LU7.

Approved Drug	Lowest Free Binding Energy to the Active Site (kcal mol^−1^)
**HIV-1 protease inhibitors**	
Lopinavir	−8.65 (2nd)
Ritonavir	−8.96 (2nd)
**HCV protease inhibitors**	
Telaprevir	−9.23
Boceprevir	−9.16 (2nd)
**DPP-4 inhibitor**	
Sitagliptin	−8.80
**Thrombin inhibitors**	
Argatroban	−9.03
Dabigatran	−6.57
**Factor Xa inhibitor**	
Rivaroxaban	−7.97
**ACE inhibitor**	
Captopril	−5.17
**Renin inhibitor**	
Aliskiren	−4.66

**Table 3:** Estimated binding energies of approved or under-trial III protease inhibitors to the structure of the SARS-CoV-2 main protease structures (PDB code: 6LU7 and PDB code: 6M2N), to their initial protease target (HCV protease structure 2WF8, DPP-4 structure 2FFW, a-thrombin structure 1DWE, Factor Xa structure 4BTI) and to the HIV-1 protease structure 4RVJ.

HCV Protease Inhibitors	Est. Free Binding Energy (kcal/mol)				DPP-4 Inhibitors	Est. Free Binding Energy (kcal/mol)			
	SARS-CoV-2		HCV	HIV-1		SARS-CoV-2		DPP-4	HIV-1
	6LU7	6M2N	2WF8	4RVJ		6LU7	6M2N	2FFW	4RVJ
Asunaprevir	−7.77	−7.52			Alogliptin	−7.42	−7.19	−9.14	
Danoprevir	−8.05	−8.00	−11.53	−7.16	Anagliptin	−8.05	−8.20	−9.16	
Faldaprevir	−10.92	−11.15	−11.27	−6.02	Evogliptin	−8.38	−8.74	−9.84	
Narlaprevir	−5.70	−6.11	−12.38	−6.90	Gosogliptin	−8.30	−8.75	−9.18	
Sovaprevir	−8.17	−8.53	−10.45	−4.80	Gemigliptin	−8.99	−9.95 −9.94 *	−10.18	−7.86
Vaniprevir	−7.12	−7.44			Linagliptin	−9.48	−8.25 −8.35 *	−9.52	−2.20
2WF8 initial ligand			−12.42		Melogliptin	−8.06	−7.01	−9.00	
4RVJ initial ligand				−9.86	Omarigliptin	−8.69	−7.91	−10.02	
6M2N initial ligand		−7.45			Saxagliptin	−8.67	−8.14	−8.28	
N3 inhibitor		−7.41			Teneligliptin	−9.58	−9.16 −9.52 *	−9.87	−6.55
					Trelagliptin	−8.92	−8.41	−9.30	
					Vildagliptin	−8.55	−8.21	−8.67	
					4FFW initial ligand (sitagliptin)			−10.78	
**α-Thrombin Inhibitors**	**Est. Free Binding Energy (kcal/mol)**				**Factor Xa Inhibitors**	**Est. Free Binding Energy (kcal/mol)**			
	**SARS-CoV-2**		**thrombin**			**SARS-CoV-2**		**FXa**	
	**6LU7**	**6M2N**	**1DWE**			**6LU7**	**6M2N**	**4BTI**	
Inogatran	−10.30 (3^rd^)	−8.32	−10.39		Apixaban	−7.53	−7.11		
Melagatran	−8.64	−8.70	−10.35		Betrixaban	−9.25	−8.44	−8.76	
1DWE initial ligand			−9.00		Edoxaban	−10.51 (2nd)	−9.11	−11.59	
					Otamixaban	−7.27	−7.33		

* Docking analysis was carried out on a molecular docking server using Autodock 4.2 via Ligandscout.

**Table 4:** Approval/trial status and side effect of the evaluated drugs.

Inhibitors		CheMBL ID	Phase	Industry	Most Common Side Effects
**HCV protease inhibitors**	**Asunaprevir**	ChEMBL2105735	Phase III clinical trials [41]	Bristol-Myers Squibb	Generally well tolerated, increased ALT/AST [42]
	**Danoprevir**	ChEMBL2311191	Approved in China 2018	Ascletis by Roche	-
	**Faldaprevir**	ChEMBL1241348	Phase III clinical trials in 2011	Boehringer-Ingelheim	Gastrointestinal events [43]
	**Narlaprevir**	ChEMBL1255891	Approved	Schering, ℞-Pharm	Pregnancy lactation, severe neutropenia [44]
	**Sovaprevir**	ChEMBL2105750	Investigational, received Fast Track status from FDA in 2012	Achillion Pharmaceuticals	-
	**Telaprevir**	ChEMBL231813	Approved 2011	Vertex Pharmaceuticals and Johnson & Johnson	Rash, anemia, leukopenia/neutropenia [44]
	**Vaniprevir**	ChEMBL599872	Approved Japan 2014	Merck & Co.	Diarrhea, nausea [45]
**DPP-4 inhibitors**	**Alogliptin**	ChEMBL376359	Approved 2013	Takeda Pharmaceutical Company	Increased risk of heart failure [46]
	**Anagliptin**	-	Approved in Japan 2012	Sanwa Kagaku Kenkyusho	-
	**Dutogliptin**	-	Phase III	Phenomix Corporation	Not known yet
	**Evogliptin**	ChEMBL1779710	Approved 2015	Dong-A ST	Headache, nasopharyngitis, upper respiratory tract infection [47]
	**Gemigliptin**	-	Approved 2011	LG Life Sciences	Generally well tolerated
	**Gosogliptin**	-	Approved in Russia 2016	Pfizer	Hypoglycemia [48]
	**Linagliptin**	ChEMBL237500	Approved 2011	Eli Lilly and Company and Boehringer Ingelheim	Angioedema, pancreatitis, joint pain [49]
	**Melogliptin**	-	Phase III	Glenmark Pharmaceuticals and Merck KGaA	Not known yet
	**Omarigliptin**	ChEMBL2105762	Approved in Japan 2012	Merck & Co.	Generally well tolerated
	**Saxagliptin**	ChEMBL385517	Approved 2009	Bristol-Myers Squibb; AstraZeneca	Upper respiratory tract infection, may cause joint pain [49,50]
	**Sitagliptin**	ChEMBL1422	Approved 2006	Merck & Co.	Headache, swelling of the legs, upper respiratory tract infection [51]
	**Teneligliptin**	-	Approved in Japan 2012	Mitsubishi Tanabe Pharma	Generally well tolerated
	**Trelagliptin**	-	Approved in Japan 2015	Takeda	Generally well tolerated
	**Vildagliptin**	ChEMBL142703	Approved 2007	Novartis	Nausea, hypoglycemia, headache, dizziness [52]
**α-Thrombin inhibitors**	**Argatroban**	ChEMBL1166	Approved 2002	Eagle Pharmaceuticals	Bleeding from the bladder, blurred vision, chest pain, dizziness, fever [53]
	**Inogatran**	ChEMBL114715	Approved 2016	AstraZeneca	Well tolerated
	**Melagatran**			AstraZeneca	
	**Dabigatran**	ChEMBL539697	Approved 2010	Boehringer-Ingelheim	Gastrointestinal [54]
	**Apixaban**	ChEMBL231779	Approved 2014	Bristol-Myers Squibb and Pfizer	Bleeding, bausea [55]
	**Betrixaban**	ChEMBL512351	Approved 2017	Millennium Pharmaceuticals, Merk	Bleeding [56]
	**Edoxaban**	CHEBI:85973	Approved 2015	Daiichi Sankyo	Stomach ache, abnormal results of blood tests that measure liver function, anemia [57]
	**Otamixaban**	-	Ended at phase III	Sanofi	-

## Abbreviations

RDRP	RNA-dependent RNA polymerase
DPP-4	dipeptidyl peptidase-4
